# Sleep and stress as modifiable drivers of Alzheimer’s disease

**DOI:** 10.1038/s44400-026-00127-2

**Published:** 2026-09-02

**Authors:** Darcy Wear, S. Abid Hussaini, Wai Haung Yu

**Affiliations:** 1 Department of Pharmacology and Toxicology, University of Toronto, Toronto, ON Canada; 2 Center for Dementia Research, The Nathan S. Kline Institute for Psychiatric Research, Orangeburg, NY USA

**Keywords:** Diseases, Neurology, Neuroscience

## Abstract

Proper sleep and stress maintenance are essential for health and may contribute to disease progression when dysregulated. This review highlights the intricate links between sleep, stress, and Alzheimer’s disease, including underlying neural circuitry and mechanisms that contribute to disease. Sleep is essential for protein clearance pathways including autophagy and the glymphatic system, which are impaired in neurodegenerative diseases. Similarly, chronic stress incites neuroinflammatory responses known to be elevated in brains of patients with neurodegeneration. As a result, it is perhaps unsurprising that tau pathology and neurodegeneration affect sleep and stress circuitry in the brain, including the Locus Coeruleus and Lateral Hypothalamus, beginning in prodromal phases of Alzheimer’s disease. Though the precise mechanisms underscoring these vulnerabilities are incompletely understood, this provides a unique therapeutic opportunity for targeting these pathways in early Alzheimer’s disease patients with concomitant sleep impairments and/or chronic stress. In this review, we offer insights into these relationships including specific brain region involvements, the contribution of molecular mechanisms of disease, and these translational opportunities which may arise.

## Part 1: Biology of Sleep, Stress, and Neurodegeneration

### Sleep stages and AD pathology

**Fig. 1:**
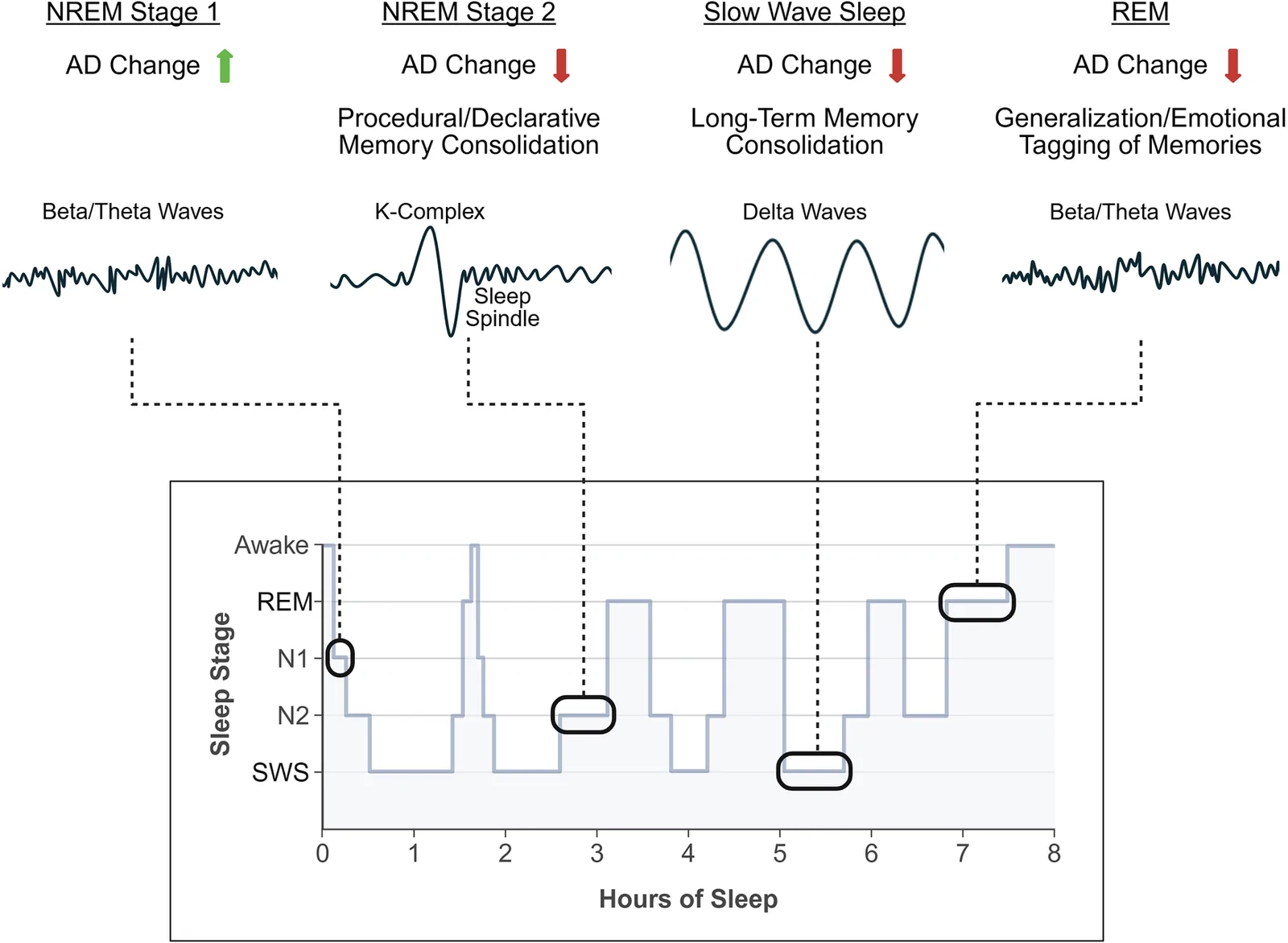
Sleep staging and importance for memory. NREM stage 1 is characterized by beta and theta waves in EEG and is elevated in AD. NREM stage 2 contains K-complexes and sleep spindles in EEG, is reduced in AD, and is important for procedural and declarative memory consolidation. Slow wave sleep is dominated by delta waves in EEG, declines in AD, and is essential for long-term memory consolidation. REM sleep appears similar to wakefulness and contains beta and theta waves in EEG, is reduced in AD, and is necessary for the generalization and emotional tagging of memories. Figure created with BioRender.com.

Sleep serves an important homeostatic role, and maintaining proper sleep hygiene is crucial for brain health. Sleep can be broken down into rapid eye movement (REM) and three stages of non-REM (NREM) sleep (Fig. 1). In light sleep or NREM stage 1, neuronal activity slows, with both wake-associated beta waves and theta waves observable in an electroencephalogram (EEG)^1,2^. As the body shifts into deeper sleep and NREM stage 2, characteristic rapid neuronal firing or sleep spindles are essential for procedural and declarative memory consolidation^3^. Low-frequency, high-amplitude long delta waves or K-complexes appear in NREM stage 2, supporting sleep maintenance and memory consolidation^2^. In NREM stage 3 or slow wave sleep (SWS), delta waves dominate, and many critical physiological functions are carried out, including increased removal of toxic proteins via the glymphatic system^4^, preserving neuronal activity by reducing synaptic strength, and active systems consolidation of long-term memories^1,5^. Bouts of REM sleep occur with dominant theta and beta waves that contribute to the generalization and emotional tagging of memories^1,2,6^.

In Alzheimer’s disease (AD), sleep-stage-related alterations appear including prolonged periods of wakefulness, increased NREM stage 1, and diminished NREM stage 2, SWS, and REM sleep. Given the latter are critical stages for toxic protein clearance and memory consolidation, this can promote the development of AD pathologies including cognitive deficits, proteostasis impairments, and proteinopathies, which further contribute to REM and NREM sleep deficits^1,7–9^. Changes in REM sleep can promote neurodegeneration, amyloid deposition, and reduced gray matter volume in the frontotemporal region of the brain^10^. Sleep disruptions induce significant changes to many of the physiological processes in AD progression. For example, a loss of REM sleep enhances norepinephrine levels, leading to reduced calcium influx and potential autophagic-lysosomal deficits, driving brain pathology^11^. Xie et al. found that chronic sleep fragmentation in wild-type mice resulted in autophagic deficiencies, anxiety-like behaviors, and microglial-induced inflammation^12^. The neuroinflammatory consequences of sleep deprivation are further exacerbated by the release of pro-inflammatory cytokines in the hippocampus (HPC) and onset of reactive astrogliosis in sleep-promoting areas linked to poor sleep in an AD pathology mouse model^13,14^.

### AD neuropathology

The complexity of AD neuropathology is highlighted by multiple underlying processes that contribute to the acceleration of disease progression. Neuroinflammatory responses by microglia and astrocytes including cytokine release, crucial for extended bouts of neuroinflammation, contribute to AD-associated proteinopathy and neurotoxicity^15,16^. Cellular perturbations such as deficiencies in homeostatic protein clearance mechanisms in neurodegenerative diseases, including the autophagic-lysosomal system (A-LS) and the ubiquitin proteasome systems (UPS), can promote the accumulation of aberrant protein aggregates^17,18^. Age-associated homeostatic impairments, including stress, neuroinflammatory, and proteostasis pathways, enhance the risk of accumulating these biological changes, resulting in aging as a primary risk factor for developing AD^19^.

β-amyloid (Aβ) plaques and neurofibrillary tau tangles remain the canonical proteinopathies of AD^20^, but co-pathologies including transactive response DNA binding protein of 43 kDa (TDP-43) and α-synuclein can also contribute to AD progression^21,22^. The amyloid precursor protein (APP) naturally contributes to neural homeostasis including developmental neurogenesis and synaptogenesis^23,24^, but Aβ formation and APP overexpression-induced reactive oxygen species impair neural function and contribute to neurotoxicity^25–27^. Impeded neural functionality alongside synaptic deficits highlight the role of Aβ in the onset and progression of AD. Pathological amyloid progression in AD has been staged by Thal and colleagues; phase 1 is characterized by neocortical Aβ deposits^28^. β-amyloid pathology proceeds to the entorhinal cortex and HPC (Thal phase 2), and the striatum, brainstem, and cerebellum in Thal phases 3-5.

The microtubule-associated protein tau is involved in microtubule structure and stability^29^. Hyperphosphorylation of tau drives intraneuronal neurofibrillary tangles formation as seen in AD and other dementias^30^. These tangles impede microtubules and axoplasmic transport disrupting neuronal functionality, with the neurofibrillary tangle burden strongly linked to dementia progression^31^. In contrast to amyloid pathology, the pathological spread of tau described by Braak and Braak begins with a pre-cortical stage (*e.g*., locus coeruleus (LC)) in Braak stage 0. In stages 1 and 2, tau is seen in the transentorhinal cortex, with entorhinal cortex spread in stages 3/4, and the HPC and neocortical areas in last 2 stages^32,33^. Notably, the initial localization of tau pathology in the LC, home to noradrenergic neurons involved in wakefulness, “fight-or-flight” response, and memory regulation, points to a potential link between sleep, stress, and AD^1,34^.

### Impact of stress in AD and sleep impairment

Increasing evidence suggests that neuropsychiatric symptoms including stress, anxiety, and depression, are modifiable risk factors of AD^35^. One study notes that both clinical depression and sleep disturbances enhance AD risk, with an additive risk effect alongside the ApoE4 carriers^36^. A potential mechanism involves prefrontal cortical astrocytes that are critical to depression development and whose function is altered by stress, leading to both glial and neuronal loss^37,38^. Through meta-analysis, Kuring and colleagues, identified that depression and anxiety were comorbidities or risk factors for dementia^39^. Consistent with this, Harerimana and colleagues found a causal genetic role for depression in AD, with 53 depression-predisposing genetic variants associated with cognitive trajectory or AD^40^. As reviewed by Botto et al., potential explanations linking mental health and AD include depression or anxiety development from coping difficulties with an AD diagnosis, neurodegeneration in neural circuitry regulating emotionality, (e.g., anterior cingulate cortex), or cognitive deficits affecting emotional responses^41^. Stress also influences mental health and is strongly linked with the onset of late-life depression. In particular, stress caused by events that incite cognitive or functional limitations, such as the onset of a neurodegenerative disease^42,43^. The Lancet Commission (2024) identified depression and social isolation (often associated with anxiety/depression) as modifiable risk factors of dementia^35^. However, sleep impairment was omitted, as further clarification on the exact sleep risk (length of sleep, sleep quality, circadian disturbances) is needed to determine causation.

**Fig. 2:**
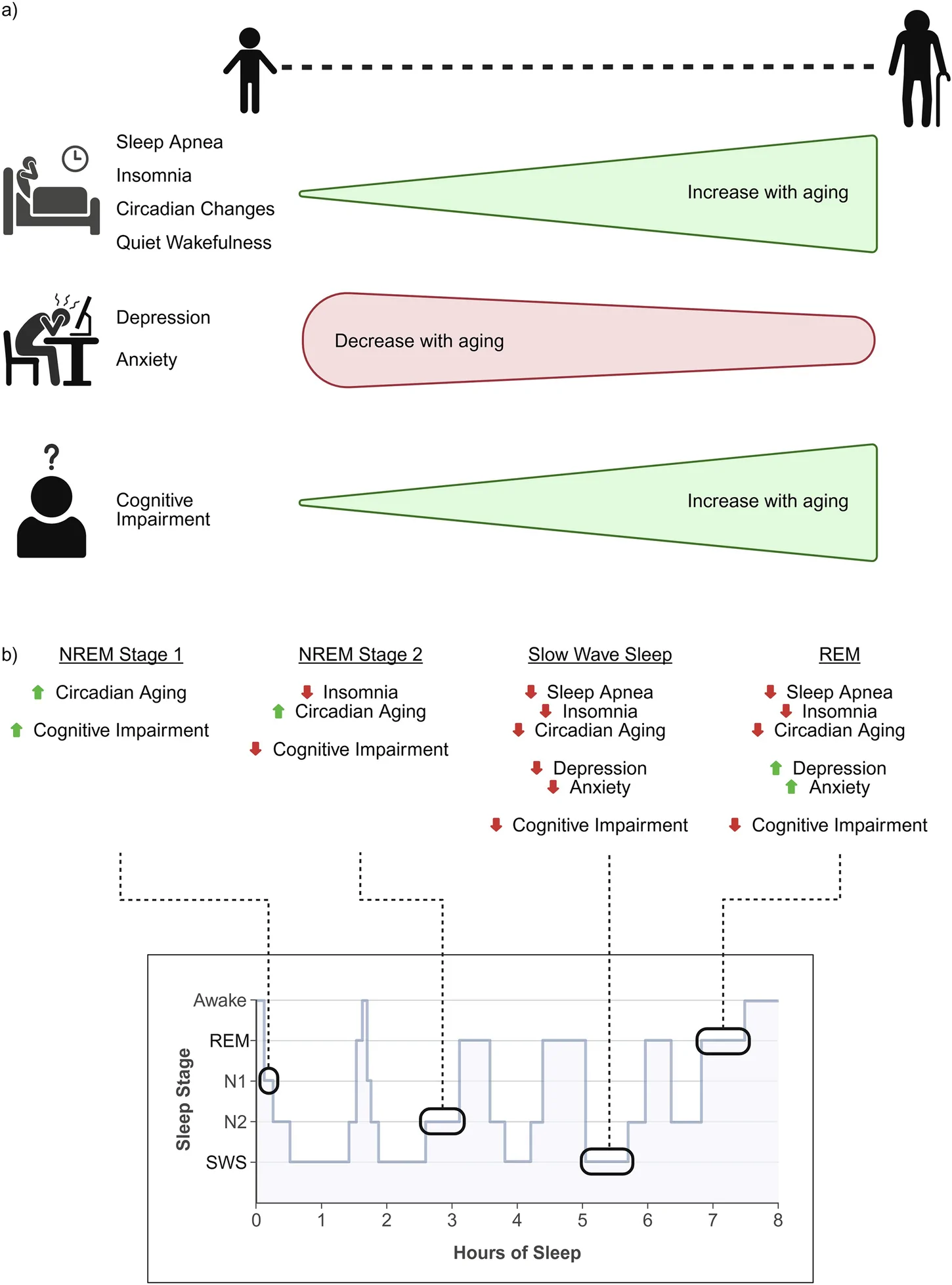
Effect of aging on sleep, stress, and cognitive disorders. **a** Sleep disorders including sleep apnea and insomnia, along with circadian changes and increased quiet wakefulness are more common with age. Conversely, depression and anxiety disorders are more common in early adulthood^51,52^ while aging is a risk factor for cognitive impairment. **b** The impact of sleep disorders, stress disorders, and cognitive impairment on each sleep stage is shown. Sleep disorders are generally associated with reduced SWS and REM sleep, while anxiety and depression reduced SWS but often increase REM sleep. Figure created with BioRender.com.

Established links between sleep and AD, as well as AD and mental health, support observed associations between sleep and mental health. Depression is an important risk factor for the onset of sleep disorders, including insomnia, particularly in women^44,45^. Given the close interrelationship of sleep and stress, it is difficult to disentangle the two and subsequently, chronic uninterrupted stress models often employ a sleep-disruption paradigm. A study by Murack et al. investigated chronic 8-day sleep disruption in CD-1 mice and observed a significant increase in depressive-like behaviors and markers^46^. Similar results have been observed in human studies across demographic cohorts including school age youth with insomnia displaying anxiety-like symptoms^47^, women with polycystic ovary syndrome whose sleep habits were impacted by the COVID-19 lockdown and displaying elevated levels of depression and stress^48^, to sleep-disrupted United States Navy personnel with increased risk of developing post-traumatic stress disorder, generalized anxiety disorder, or major depressive disorder^49^. Although causality cannot be inferred from these studies, there is a consistent association between individuals with disrupted sleep and those experiencing anxiety and/or depression. Meta-analyses have associated causality by examining the extent to which sleep improving interventions affected mental health. Dose-dependent improvements in measures of depression, anxiety, and stress highlight the importance of sleep in mental health^50^. The incidence of sleep and stress disorders with aging, along with their impact on sleep architecture, is summarized in Fig. 2^51,52^.

### Sex Differences in AD and Sleep

Sex is a biological variable that is critical to understanding AD. Women have significantly higher rates of AD, with an estimated lifetime risk of 21.1% versus 11.6% in men at age 65, and present with increased levels of pathology^53–56^. Behavioral, imaging, and post-mortem studies support these sex differences in AD. Females demonstrate reduced cognitive performance and brain volumes with AD pathology than males^56^, have increased susceptibility to entorhinal cortical tauopathy^57^, have greater mitochondrial impairments in late-onset AD^58^, and display elevated frontal cortex tau hyperphosphorylation in post-mortem tissue from AD patients with psychoses^59^. Preclinical pathological differences including elevated amyloid plaques, neurofibrillary tangles, and associated neuroinflammation in female triple transgenic AD mice (APP/PS1/tau) were shown by Yang and colleagues^60^. Similarly, in a progressive amyloid mouse model of AD, studies have shown increased astrogliosis and amyloidosis in aged females^61^, enhanced severity of neuropathological changes including neuroinflammation, synaptic deficits, and β-amyloid deposition, in young females^62^, and increased microglial activation in response to amyloid in females^63^.

Sleep, stress, and dementia are intricately intertwined, with additional sex differences highlighting the complexities of this dynamic. The prevalence of sleep-disrupting conditions varies between sex with women more likely to develop insomnia, and sleep apnea more common in men^64,65^, though there is an approximately 2% increase in rates of sleep apnea in post-menopausal women^66^. Although insomnia is more prevalent in women, men with insomnia experience significantly increased risk of cognitive decline, but not women^67^. Similarly, women with sleep apnea are more likely to develop dementia than men, suggesting unique sex-based mechanistic vulnerabilities to sleep impairments^45,68^. Sleep-related sex-differences have been reported in pre-clinical AD models including 5XFAD mice, where only female mice show reduced total sleep (versus wild-type), and in APP^NL-G-F^xMAPT double knock-in (KI) mice, where females displayed more rapid sleep impairments, including reduced REM sleep^69,70^. Stress responses in AD show additional sex differences in human APP-KI mice with female but not male Aβ levels increased by acute stress, mediated through β-arrestin internalization of the corticotropin-releasing factor receptor. In males, β-arrestin internalizes the receptor during stress to block the upregulation of γ-secretase and subsequent increase in β-amyloid^71^.

Male and female rodents exhibit unique vulnerabilities to chronic mild stress. Dalla and colleagues showed increased susceptibility to chronic mild stress in female rats, including reduced serotonergic activity in the HPC and LHA and elevated serum corticosterone^72^. Interestingly, females responded better to a subsequent novel stressor with elevated serotonergic activity in the LHA versus males, while males showed elevated corticosterone levels only after the second novel stressor versus control^67^. Another study reported males were more vulnerable to somatic effects, including changes to heart rate and body weight, while females experience neuroendocrine and behavioral changes, including elevated corticosterone^73^. These data may translate to humans where women are more likely to experience stress-related psychiatric disorders, including depression and PTSD^74,75^. Future research examining sleep-stress-AD must account for sex and gender to accurately assess disease progression, including behavioural and biochemical changes. Investigating these differences will provide insight into AD heterogeneity and support the development of targeted precision medicine approaches.

## Part 2: Neural Basis for the Sleep-Stress-AD Relationship

**Fig. 3:**
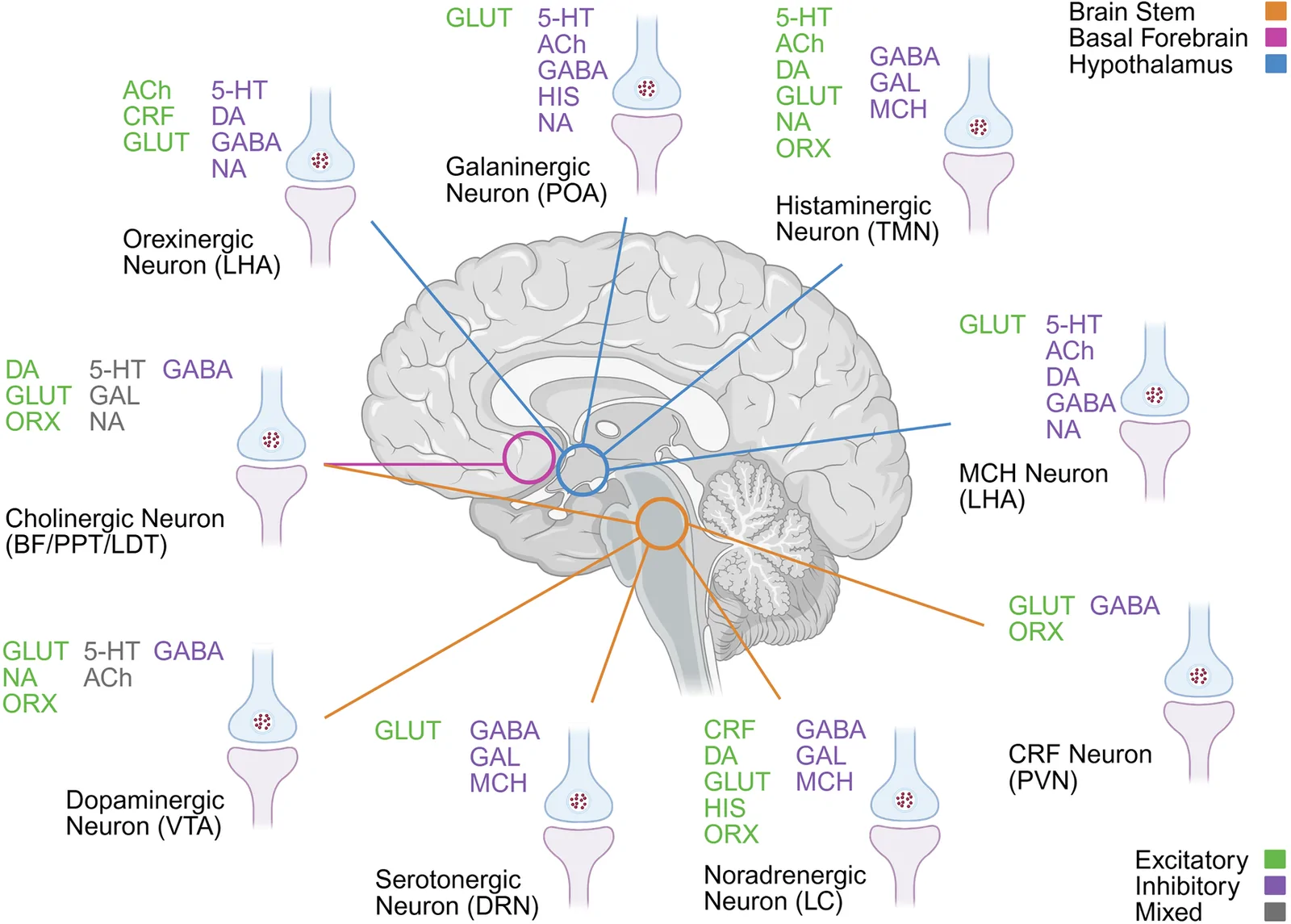
Primary sleep and stress regulatory neural synapses. Pre- (blue) and post-synaptic (pink) neurons of the brain stem, basal forebrain, and hypothalamus shown with primary neuron types, as well as neurotransmitter inputs (excitatory – green, inhibitory – purple, excitatory or inhibitory – gray). 5-HT Serotonin, ACh Acetylcholine, CRF Corticotropin Releasing Factor, DA Dopamine, GABA Gamma-Aminobutyric Acid, GAL Galaninergic, GLUT Glutamate, HIS Histamine, MCH Melanin-Concentrating Hormone, NA Noradrenaline, ORXs Orexin. Figure created with BioRender.com.

Sleep, stress, and dementia are intricately linked via neural pathways. These complex networks include multiple neuronal subtypes with distinct functions to achieve balance in sleep/wake and stress responses (Fig. 3). As an example, the orexinergic signaling pathway of the lateral hypothalamus (LHA) is particularly vulnerable prodromal to and in very early AD^76^. Orexinergic neurons project to noradrenergic (NA) neurons in the LC and corticotropin-releasing-factor (CRF) neurons of the paraventricular nucleus (PVN) coinciding with their role in sleep and stress^77,78^. NA neurons have minimal firing during sleep and incite wakefulness in response to orexinergic signaling^1,79^. CRF neurons of the PVN serve as master regulators of the hypothalamic-pituitary-axis (HPA), crucial for cortisol release and the fight-or-flight stress response^80,81^. Recent evidence suggests impairments to the autophagic-lysosomal system in these vulnerable neuronal populations, particularly the LC and LHA, may be critical to AD progression^69,76,82^. Furthermore, excitation-inhibition imbalances in AD neurocircuitry can incite hyperexcitability and is essential to AD pathology^83^.

Two of the primary sleep/stress regulating brain regions are the brain stem and the hypothalamus. The brain stem contains several neuronal subtypes involved in sleep and stress regulation including wake-promoting cholinergic, dopaminergic, serotonergic, and noradrenergic neurons, as well as stress-regulating corticotropin-releasing factor-expressing neurons. Similarly, the hypothalamus is home to wake-promoting orexinergic and histaminergic neurons, along with sleep-promoting galaninergic and melanin-concentrating hormone (MCH) neurons. These populations are intricately intertwined, contributing excitatory and inhibitory synapses onto one another, summarized in Fig. 3, to ensure proper sleep/wake regulation is maintained. In this section, we will review the major cell types involved in these networks, highlighting functions and alterations or contributions to disease.

**Serotonergic neurons** of the dorsal raphe nucleus (DRN) contribute to wakefulness, largely by inhibition of REM sleep, and response to uncontrollable stress^84,85^. They receive inhibitory inputs from melanin-concentrating hormone (MCH) hypothalamic neurons, GABAergic and galaninergic neurons to modulate sleep-wake control^1,86^. Serotonergic efferents project to many regions, including the thalamus, hypothalamus, prefrontal cortex (PFC), basal forebrain (BF), amygdala, HPC, ventral tegmental area (VTA), and the REM-generating cholinergic neurons of the pedunculopontine and laterodorsal tegmental nuclei (PPT/LDT)^1,84^. Outputs to the amygdala and VTA regulate emotional health including anxiety, reward, and motivation^87–89^. Similarly, abnormal serotonergic signaling contributes to CA1 pyramidal hyperexcitability in AD, with glutamatergic hippocampal neurons governing cognition and depressive symptoms^90–92^.

**GABAergic interneurons**, located throughout the brain, promote sleep and are active during NREM sleep^1^. Several subtypes including parvalbumin, calretinin, somatostatin, cholecystokinin, neuropeptide Y, and vasoactive intestinal peptide neurons are involved in the regulation of sleep^93^. One of these primary GABAergic populations lies in the VLPO, and receive sleep-blocking signals from cholinergic, NA, serotonergic, and histaminergic populations^86,94^. Conversely, they block wake-promoting neurons including the orexinergic and histaminergic neurons of the hypothalamus, NA neurons of the LC, serotonergic neurons of the DRN, and glutamatergic neurons of the parabrachial nucleus^1,95^. GABAergic signaling from the parafacial zone of the medulla is crucial for SWS through parabrachial glutamatergic inhibition^96^. Some exceptions exist with wake- and REM-active GABAergic neurons, including the aforementioned parvalbumin neurons of the BF, and GABAergic populations in the lateral hypothalamus^1,97,98^. As with excitatory glutamatergic neurons, the inhibitory GABAergic populations are important to maintain an excitation-inhibition equilibrium, with a loss of GABA expression associated with chronic stress^99^.

In AD, various GABAergic deficits occur including reduced hippocampal GABAergic signaling^100,101^, reduced expression of GABA receptor-related genes^102^, and increased vulnerability of somatostatin GABAergic neurons associated with cognitive impairment^103,104^. AD mice also have an age-dependent parvalbumin GABA_A_ receptor loss increasing anxiety levels^105^. BACE1-induced cleavage of this GABA_A_ receptor promotes hyperexcitability in the HPC impairing cognition and contributing to neurodegeneration^106^. In the cortex, GABA loss impedes slow oscillations essential for memory consolidation during sleep and may be important for Aβ accumulation in AD^107^. Indeed, Zhao and colleagues found impaired NREM sleep and increased wakefulness in APPswe/PS1dE9 mice, consistent with aberrant slow oscillations, which promoted AD progression. However, optogenetic stimulation of cortical GABAergic interneurons enhanced slow-wave activity, rescuing sleep impairments and slowing AD progression and Aβ pathology^108^.

**Galaninergic neurons** of the preoptic area promote sleep via inhibition of arousal systems including NA neurons of the LC, serotonergic neurons of the DRN, histaminergic neurons of the hypothalamus, and cholinergic neurons of the BF^1,86,109^. Conversely, arousal signals from NA and acetylcholine inhibit galaninergic neurons directly and indirectly, as they activate GABAergic neurons in the preoptic area to further suppress galaninergic neuron activity^110^. In AD, galaninergic signaling is paradoxical and complex. AD patients experiencing sleep fragmentation have galaninergic neuron loss correlating with the magnitude of sleep impairment^111^. However, both galanin and its receptors show increased expression in cognition-associated brain regions. This upregulation could target and inhibit cholinergic neurons of the BF resulting in cognitive impairment in spatial navigation and olfactory memory tests^109^. This hyperinnervation of BF cholinergic neurons may promote neuronal function and survival, delaying AD symptoms^112^.

**Noradrenergic neurons** of the LC play a central role in arousal, wakefulness, and memory regulation^1,113,114^, receiving inhibitory inputs from GABAergic neurons of the ventrolateral preoptic area (VLPO) and median preoptic nucleus, galaninergic neurons, and MCH neurons, reducing firing during sleep^1,86^. Conversely, excitatory inputs from orexinergic and histaminergic neurons promote wakefulness, while indirect projections from the suprachiasmatic nucleus (via dorsomedial hypothalamus) modulate circadian rhythm regulation of arousal^1,86,115^. Outputs from these noradrenergic neurons inhibit sleep-promoting neurons in the VLPO and promote excitation of pyramidal neurons of the PFC^116,117^. In AD, these neurons are amongst the earliest with tauopathy, and may play a role in tau spread to the neocortex and HPC^33^. In turn, tau-induced hyperexcitability of NA neurons may dysregulate the sleep-wake balance and incite neuroinflammatory consequences^118,119^. Due to their crucial nature in modulating glymphatic clearance during NREM sleep, loss of these neurons likely contributes to proteostasis dysregulation in AD^120^.

**Orexinergic, histaminergic, and MCH neurons** of the hypothalamus balance sleep-wake regulation, with orexinergic and histaminergic signaling important for arousal/wakefulness, while MCH neurons induce sleep^1,121^. These hypothalamic inputs are also important in stress and anxiety response, via orexinergic projections to the CRF-neurons and MCH projections to the basolateral amygdala^77,122,123^. Furthermore, increased brain histamine from acute stress indicates a role for histaminergic neurons in stress responses^123,124^. These neurons receive inputs from noradrenergic, serotonergic, dopaminergic, and cholinergic neurons of the brain stem and target various brain regions including the LC, PVN, HPC, and BF^1,84,86,125,126^. Early in AD, tauopathy in the hypothalamus leads to loss of wake-promoting orexinergic and histaminergic neuron populations, with orexinergic neuron loss preceding even LC tauopathy. However, sleep-promoting MCH neurons display resilience to tau pathology thereby upsetting the intricate sleep-wake balance^1,76,127^. APP^NL-G-F^ mice have reduced MCH neuronal activation inciting sleep-wake impairments and Aβ-induced hyperexcitation of hippocampal CA1 neurons^128^. In humans, CSF levels of MCH are upregulated in AD and correlate with pathological measures including reduced cognition, and total and p-Tau CSF levels^129^. Similarly, despite reduced orexinergic neurons, AD patients exhibit increased orexin-associated gene expression and elevated CSF levels of orexin, likely compensating for dysregulated sleep mechanisms^1,76^. This increase in CSF orexin is important for thalamic and amygdala atrophy mediating depression in AD patients^130^.

**Cholinergic neurons** of the BF are major sleep regulators promoting wakefulness and REM sleep, while those of the PPT impair low-frequency EEG rhythms in NREM and increase the light to deep NREM sleep ratio^1,86,131^. Complex cholinergic BF circuitry includes excitatory wake-promoting signals from the orexinergic neurons, excitatory input from parabrachial glutamatergic neurons, excitatory and receptor subtype-specific inhibitory serotonergic signals, and inhibitory sleep-promoting signals from somatostatin neurons^1,84,86,132,133^. PPT cholinergic neurons are signaled to by afferents from the superior colliculus, substantia nigra, striatum, and brainstem motor nuclei^134^. Similarly, LDT cholinergic neurons receive input from the superior colliculus, substantia nigra, striatum, and the limbic cortical areas - DRN and LC. These circuits involve dopaminergic, serotonergic, and GABAergic signaling to the PPT and LDT^134^. Linked with their additional roles in memory and attention, BF cholinergic neurons innervate the cortex, HPC, and amygdala, exciting pyramidal neurons and driving theta activity^84,135^. Unique BF populations innervate the anterior cingulate cortex to target an excitatory isocortical centre, or innervate the basolateral amygdala to target inhibitory centres in the striatum and pallidum^136^. Cholinergic neurons in the LDT/PPT primarily project to the thalamus and BF with low cortical innervation^1,84,135^. In early AD, cholinergic neuron loss and BF atrophy are linked to cortical thinning and cognitive decline, a relationship enhanced by increased Aβ^137^. This may be, in part, a result of the links between cholinergic signaling and inflammatory glial responses, Aβ accumulation, and synaptic health^138^. Furthermore, the accumulation of toxic tau oligomers in these vulnerable cholinergic neurons in early AD contributes to neuronal impairments^139^. Highlighting its importance in the sleep-AD relationship, reduced cholinergic signaling in AD impacts EEG complexity, with cholinergic BF compensatory mechanisms improving sleep architecture and hippocampal REM activity in an early-plaque stage AD rat model^140,141^.

**Glutamatergic neurons**, located throughout the brain are associated with wakefulness and REM sleep. Within the BF, these neurons promote wakefulness through innervation of neighboring excitatory cholinergic and parvalbumin GABAergic neurons, and are inhibited by somatostatin neurons^86,135^. This interplay between the excitatory wake-promoting glutamatergic, parvalbumin GABAergic, and cholinergic neurons, and the inhibitory somatostatin GABAergic neurons highlight sleep-wake complexity in this region^1^. These glutamatergic neurons also induce cortical activation and firing during wake and REM sleep^86,135^. Similarly, glutamatergic neurons in the supramammillary nucleus (hypothalamus) project to the BF, cerebral cortex, and HPC, inducing wake and REM sleep^95,142^.

Several brainstem nuclei contain glutamatergic populations as well, including the parabrachial nucleus, important for promoting the wake-state, as well as the PPT and LDT, which are important for REM sleep and wakefulness^1,135^. The sublaterodorsal nucleus of the pons contains strictly REM-promoting glutamatergic neurons inhibited by GABAergic neurons in the ventrolateral periaqueductal gray region during NREM sleep and wake^86,95,135^. This glutamatergic signaling leads to muscle paralysis during REM sleep by the activation of inhibitory interneurons in the medulla and spinal cord^95,135^. Uniquely, a glutamatergic neuronal subpopulation in the dorsolateral pons promotes NREM sleep and strongly suppresses both wakefulness and REM sleep^86^.

Glutamatergic signaling is instrumental for acute and chronic stress responses, and has been reviewed previously^143,144^. Briefly, acute stress enhances glutamate release (PFC, HPC, amygdala) improving synaptic function. However, chronic stress results in excessive glutamate signaling inducing detrimental effects including excitotoxicity and a loss of dendritic branching in the PFC^143,144^. In AD, this excitotoxicity is exacerbated by extracellular Aβ which limits astrocytic glutamate uptake, further depolarizing neurons and promoting a positive feedback loop of hyperactivity^145,146^. Reduced levels of glutamate in cortical regions of AD brains further highlights the imbalance in glutamatergic signaling with disease and importance of glutamate in AD^147,148^. Furthermore, the AD risk gene BIN1 regulates gene expression in these glutamatergic neurons^149^.

**Dopaminergic neurons** (VTA) are crucial for arousal and wakefulness with reduced activity during NREM sleep^150^. Through their role in regulating motivation and reward anticipation, they are involved in stress response, where both acute and chronic stress diminish reward-seeking behaviors^151^. These neurons receive modulatory inputs from the anterior cortex, orexinergic neurons, paraventricular nucleus (PVN), and serotonergic, GABAergic, and glutamatergic neurons from the DRN. Dopaminergic targets are widespread including the striatum, the nucleus accumbens, the PFC, the amygdala, and the HPC^152^. In AD, dopaminergic neuronal loss is associated with cognitive deficits and neuropsychiatric symptoms. Specifically, reduced dopamine impacts synaptic health of CA1 and CA3 neurons impeding recognition memory^153,154^. Axonal loss in the nucleus accumbens impacts reward and motivation, while novelty seeking behavior is linked to age-related decline in dopaminergic activity^155,156^. Activation of these neurons can ameliorate AD pathology by upregulating the Aβ-degrading enzyme neprilysin to reduce cortical plaques^157^.

**Corticotropin-releasing factor-expressing neurons** of the paraventricular nucleus play a key role in coordinating stress responses. They are a major orexinergic target and regulate the HPA’s stress response^80,81^. CRF neurons can directly signal neighboring CRF neurons to coordinate endocrine function^158^. Due to the inherent intricacies between stress and sleep, sleep-deprivation can modulate CRF levels, with increases in striatum, limbic structures, and the pituitary while hypothalamic CRF is reduced^159^. These neurons are important in the stress-AD relationship, including sex-differences. With CRF overexpression, females have elevated tau hyperphosphorylation, β-amyloid plaques, and cognitive impairments compared to males^71^. Further, CRF receptor 1 (CRFR1) antagonism reduced cognitive impairment in female mice only, while synaptic deficits, oxidative stress, and Aβ levels were rescued in both male and females^160,161^. However, CRFR1 may be protective in AD, as activation from a stimulating environment improves synaptic health^162^. Responding to stress, CRF and CRFR1 are important for increased interstitial fluid levels of aberrant tau and Aβ levels^163–165^. While CRF overexpression promotes pathology, levels are reduced in AD, while cortical receptor density increases. However, CRF receptor binding is further mediated by CRF-binding protein rendering free-CRF unable to bind. Consequently, CRF levels do not comprehensively explain the receptor activation^166^.

**Astrocytes** are important for the glymphatic and neuroinflammatory responses (discussed in Part 3) and are critical regulators of synaptic health. They actively communicate with neurons through tripartite synapses, resulting in a significant contribution to these neural networks underlying sleep, stress, and AD^167^. Astrocytes contribute to slow wave activity during NREM stage 3, which is impaired in AD and mouse models of amyloidosis. Furthermore, the activity of astrocytes is impaired in the same amyloidosis models^168–170^. Crucially, the restoration of this astrocytic function via optogenetic stimulation can restore sleep circuit activity and slow AD progression^168,171^. Thus, astrocytes play a crucial role in the underlying neural circuitry involved in amyloid-related sleep disturbances to NREM sleep. In response to stress, norepinephrine binds to high levels of α- and β-adrenergic receptors on astrocytes inducing an intracellular rise in calcium, promoting gliotransmitter release which modulates local synaptic plasticity^172,173^. This elevated release of gliotransmitters could exacerbate neuronal excitotoxicity observed in AD^174^.

## Part 3: Molecular Mechanisms of Disease

### Protein clearance mechanisms

Proteinopathies are a key pathological feature of neurodegenerative diseases. Many diseases, including AD, possess deficits in homeostatic protein clearance mechanisms including the A-LS, UPS, extracellular secretion, and the glymphatic system. These processes are tightly regulated by sleep and stress further supporting them as drivers of AD progression.

The **autophagic-lysosomal system** is the body’s recycling mechanism to metabolize aberrant proteins and dysfunctional organelles. The process involves the delivery of cargo to the lysosome for degradation, though the method of delivery can vary by subtype - macroautophagy, (engulfment of cargo by a double-membraned autophagosome), microautophagy (cargo uptake directly by the lysosome through invagination), and chaperone-mediated autophagy (LAMP2a receptor-mediated transport across the lysosomal membrane)^175,176^. Autophagosomes are elevated during wake and reduced during sleep in Drosophila, and this relationship is bidirectional, with upregulation of autophagosomes impairing sleep while reduction enhances sleep^177^. This could be related to efficiency of macroautophagy, as increased *Atg8a*, a gene involved in autophagosome maturation, improves circadian and sleep circuitry function in Drosophila, highlighting the complexity of this relationship^178^. What is clear is the profound impact of sleep loss on the autophagic-lysosomal system. Several studies have highlighted that sleep deprivation (SD) is detrimental to autophagy, with reduced protein clearance and toxic cargo build-up^69,179–182^. In AD, this includes the canonical proteinopathies Aβ and tau resulting in plaque and neurofibrillary tangles with SD^17,183^. In APP-KI mice, autophagic deficits appear in the sleep-regulating hypothalamus and LC prior to significant Aβ deposition outlining the dynamics of autophagy, sleep and AD progression^69,184^. Similarly, chronic stress impedes the A-LS inciting learning deficits and impacting adult HPC neurogenesis^185,186^. Furthermore, protein build-up can incite neurons to secrete cellular contents through extracellular vesicles (EVs) which may spread pathologies to neighboring and distant cells, enhancing disease progression^187^.

The **ubiquitin-proteasome system** is another key intracellular proteostatic degradation process. It involves protein-tagging of cargo with poly-ubiquitin chains, for recognition and subsequent degradation by the proteasome^188^. The UPS regulates the degradation of clock proteins and maintenance of circadian rhythm^189^. If this fine balance is impaired through UPS deficits, leading to reduced degradation of clock proteins, circadian rhythm timing becomes unstable^190^. As proteasomal deficits are common with Aβ accumulation, this could induce circadian disruptions in individuals with AD^191^. Additionally, obstructive sleep apnea (OSA) disturbs proteasomal function through intermittent hypoxia^192^, and a bidirectional relationship with chronic stress exists, including impaired UPS with chronic stress and elevated proteasome activity linked to stress-resistance in mice^193,194^. As tau levels are strongly correlated with UPS proteins and proteasomal dysfunction, dysregulation of the UPS in patients with concomitant sleep/stress disorders and AD may accelerate tau pathology and cognitive decline^18,195^.

**Extracellular vesicles** serve several functions including cell-to-cell communication, cell migration, and importantly for disease context, cargo clearance, and can be subcategorized based on size or cellular origin^187^. Although research on sleep and brain-derived EVs is limited, sleep deprivation can trigger small EV release, plus exosomes containing inflammatory signals^196,197^. While these studies are cardiovascular focused, they highlight the potential for brain-derived EVs to be pro-inflammatory and neurotoxic, contributing to AD progression. Similarly, acute and chronic stress-induced circulating EVs possess altered miRNA content which contributes to anxiety-related behaviors^198,199^. Furthermore, stress-modulating glucocorticoids have been shown to stimulate small EV release from neurons in the brain^200^. However, many brain-derived EVs originate from glial cells, including microglia which secrete miR-146a-5p-containing exosomes in response to chronic stress, which supresses neurogenesis in rats^201^. As neurogenesis is reduced in AD, this could highlight a potential link between chronic stress and AD pathology^202^. In AD, EVs have been shown to transport Aβ and increase plaque load^203–205^, while tau filaments are recently showed to be selectively packaged in EVs^206^, with inhibiting microglial EV synthesis limiting tau spread^207^. This highlights that while normally homeostatic, EV release in AD may provide a platform for the spread of proteinopathy in the brain. Furthermore, EVs can enhance recipient neuron activity highlighting a potential role in neural excitation-inhibition balance^208^. The importance of sleep impairment and stress in brain EV biology is an understudied field which may be essential to sleep loss and stress-driven AD progression.

**Glymphatic clearance** is a metabolic waste clearance mechanism involving the mixing of cerebrospinal fluid (CSF) with interstitial fluid (ISF) and clearance of soluble metabolites facilitated by aquaporin-4 (AQP4) channels in astrocytic endfeet^209,210^. This proteostasis mechanism is highly regulated through sleep, as interstitial space expands by 60% during SWS enhancing glymphatic exchange^211,212^. Furthermore, infraslow arterial oscillations driven by noradrenaline signaling from the LC promote glymphatic clearance during NREM sleep^120^. Glymphatic dysfunction occurs early in AD, likely a result of diminished time spent in SWS^213^, and altered CSF circulation and volume influencing glymphatic drainage^214^. These glymphatic impairments have been shown to increase Aβ accumulation/deposition, and promote tau aggregation^215–217^. Importantly, glymphatic activity correlates with cognitive scores^216,217^, with functional glymphatic clearance reducing mild cognitive impairment (MCI) risk and delaying progression from MCI to AD by ~3.5 years^218^. These changes are exacerbated by concomitant vascular complications in AD, that further disrupt glymphatic clearance^1^. While glymphatic clearance is an astrocytic function, astrocytes in AD are classically associated with aberrant neuroinflammation.

### Neuroinflammation

Neuroinflammation is a positive defense system including neutralizing pathogens, cell debris scavenging, and the maintenance neuronal health^219,220^. However, hyperactive sustained inflammation may significantly contribute to disease onset, including AD. In concurrence, AD patients display elevated inflammatory cytokine levels in serum and cortical brain tissue^221,222^. Genetic studies have linked several inflammation-related genes to AD including complement system components, tumour necrosis factor (TNF) signaling, and inflammasome activation^223,224^. Crucially, ~25% of identified AD risk factor genes are linked to microglial signaling or immune function, including triggering receptor expressed on myeloid cells 2 (TREM2)^223,225,226^. Neuroinflammation, including reactive microgliosis, begins at prodromal stages of AD, and may initially be protective^227^. However, glial cell-induced neuroinflammation can exacerbate oxidative stress in disease, damaging synapses and contributing to cognitive decline^228^. Furthermore, cytokine release promotes AD progression through increased tauopathy and Aβ accumulation, fomenting a positive feedback loop with neuroinflammation^229^.

The resident immune cells of the central nervous system, microglia, are elevated in AD brains^226^. These microglia characteristically surround amyloid plaques and are associated with neurofibrillary tangles, contributing to neurotoxicity^15,230,231^. Another glial cell type heavily involved in AD pathology and progression is astrocytes, normally playing a pro-survival role involving synaptogenesis and neurotransmission, as well as amyloid uptake and degradation^232–234^. However, they secrete and contain receptors for cytokines and chemokines playing a role in extended bouts of neuroinflammation and contributing to amyloid deposition under stress-inducing conditions^16,235^. In synergy, microglial cytokines induce A1 astrocytes, which are highly expressed in AD, impairing their neuroprotective influence^236^. Furthermore, astrocytes contain the components for amyloidogenic APP processing and may be involved in Aβ production in AD^237^. Like microglia, astrocytes may provide an important link between Aβ and tau pathology, as Aβ levels only associate with plasma tau in cases of positive astrocyte reactivity^238^. They have additional relevance in biomarkers as elevated CSF astroglial marker YKL-40 may be an effective predictor of poor sleep in amyloid-beta positive adults^239^.

Intertwined in the AD-neuroinflammatory dynamic are circadian rhythm, sleep, and stress. Much of our knowledge of these relationships are derived from rodent studies of sleep deprivation and chronic unpredictable stress. One study observed contributions from microglial histone acetyltransferase EP300 in sleep-impairment post-chronic unpredictable stress, alongside increased interleukin-1β^240^. Similarly, sleep restriction can induce a neurotoxic astroglia response, elevate pro-inflammatory cytokines, and incite cell senescence in the cortex and HPC^241^. These neuroinflammatory responses may partially explain sleep-deprivation-induced cognitive deficits, as treatments impede neuroinflammation and improve cognition^242,243^.

Microglial response to sleep loss and stress may be contradictory, with one study finding enhanced microglial activity and inflammatory markers associated with impaired recognition memory in wild-type mice following sleep fragmentation^244^, while another study using chronic unpredictable stress in 5xFAD mice found elevated corticosterone impeded microglial activation and Aβ uptake, with accompanying spatial memory deficits^245^. Adding circadian intricacies to this relationship, sleep deprivation in C57BL/6 J mice can upregulate serum corticosterone and clock gene expression, inducing anxiety-like behaviors and neuroinflammation while altering circadian rhythm^246^. Further supporting this fine balance, loss of circadian clock protein BMAL1 induces astrogliosis in the cortex and HPC, synaptic impairments, and oxidative damage^247^. Targeting this pathway may hold therapeutic potential given that treatment with Shuangxia Decoction, a traditional Chinese medicine used to treat sleep disruption, bound microglia activating circadian rhythm genes BMAL1 and CLOCK, leading to reduced neuroinflammation and slowed AD progression^248^. In dementia-free participants with parental AD history, proximity to parental AD onset has been shown to be associated with hypersomnia and elevated CSF neuroinflammatory biomarkers. It is plausible that longer sleep might be compensatory to neuroinflammation as they approach AD onset, and targeting AD-related sleep deficits may dampen neuroinflammatory outcomes^249^.

## Conclusions

Although not all people with AD have sleep impairments or elevated stress, we highlight their potential to accelerate AD progression, including the underlying biological mechanisms and neural circuitry. We propose the early pathological state of sleep and stress-regulatory brain regions, including the LC and LHA, in AD, signifies the importance of these processes for disease progression. This review also exemplifies the importance of precision medicine in AD therapeutics, with potentially unique therapeutic strategies for individuals with concomitant sleep impairments or stress disorders (Supplementary Information). Targeting sleep may recover the dysfunctional cellular processes in disease; alternatively, targeting impaired inputs including proteostasis or neuroinflammation, may improve sleep hygiene and consequently reduce AD risk/progression. However, mechanistic insights are still required to determine the individual contributions of different sleep impairments and how they influence neuropathology.

## Supplementary information

## Data Availability

No datasets were generated or analysed during the current study.
