# Supplementary material for: Sleep and stress as modifiable drivers of Alzheimer’s disease
Source: NPJ Dement. 2026 Sep 2;2(1):84. doi: 10.1038/s44400-026-00127-2 (PMC13538048; doi:10.1038/s44400-026-00127-2)
Supplement: Supplementary file 1 [file 44400_2026_127_MOESM1_ESM.pdf]

Sleep and Stress as Modifiable Drivers of Alzheimer's Disease

Darcy Wear[a*], S. Abid Hussaini[b], Wai Haung Yu[a*]

[a] Department of Pharmacology and Toxicology, University of Toronto, Toronto, ON, Canada

[b] s.abidhussaini@gmail.com

[*] Correspondence: haung.yu@utoronto.ca; Tel: 1-437-348-3190

d.wear@mail.utoronto.ca

Translational Opportunities

Despite the increasing prevalence of AD, current treatments focus on symptomatic relief and are limited in their ability to halt disease progression. Cholinesterase inhibitors including donepezil, galantamine, and rivastigmine are administered to people with mild to moderate AD dementia and prolong post-synaptic neural stimulation to improve signal transduction,[1] while those with more severe AD receive the NMDA receptor antagonist memantine,[2,3] which prevents glutamate binding averting mitochondrial membrane potential disruptions and excitotoxicity.[4] Maximal symptomatic relief in AD is often achieved through some combination of NMDA receptor antagonists and cholinesterase inhibitors. More recently, the anti-Aβ monoclonal antibody, Lecanemab and others, reduce amyloid markers in early AD and have cognitive benefits.[5]

Alternatively, early intervention strategies, including at prodromal states, targeting disease-promoting mechanisms may help limit the onset and progression of AD. In particular, pharmacological manipulation of sleep and stress pathways represents a promising therapeutic approach, as sleep/wake impairment is tightly linked to neuroinflammation and proteostasis deficits, known to contribute to AD pathogenesis. As these lifestyle factors are increasingly recognized as upstream contributors to AD onset, improving dysregulated sleep and stress neurocircuitry may provide an excellent approach to impeding disease development.

As noted in this review, regulation of sleep and circadian rhythm is complex involving multiple brain regions and neuronal subtypes. These complex links between sleep and dementia provide an intriguing target for the development of novel therapeutics to treat AD. For example, sleep-promoting dual orexin receptor antagonists (DORAs) like Suvorexant, Lemborexant, and Daridorexant, used to treat insomnia, could be useful as potential therapeutics to improve AD-associated symptoms and pathologies, with Suvorexant previously increasing total sleep time in a Phase III clinical trial of individuals with probable AD dementia and insomnia.[6–8] Currently, trials with these DORAs are looking at Aβ and tau pathology (NCT04629547, NCT06274528, NCT07213349). Similarly, melatonin and melatonin receptor agonists can regulate circadian rhythm, may inhibit orexinergic neurons to promote sleep, and have been tested in clinical AD trials, though they showed no impact on sleep quality or cognition.[9–12] Targeting of cannabinoid receptor 1 (CB1) with cannabidiol can promote sleep and could be another therapeutic for AD, where levels of CB1 are reduced.[13,14] This is currently in Phase II trials looking at biomarkers and clinical progression of AD with treatment (NCT05822362). Finally, non-benzodiazepine hypnotics including Zolpidem and Zopiclone enhance GABAergic signaling promoting sleep, and have been assessed in a stage 3 clinical trial on AD.[15] However, despite improving nighttime sleep measures, this trial showed worsened executive function and working memory in the treatment groups.[15] Supplementary Table 1 summarizes clinical trials involving the treatment of sleep in dementia.

Another class of drugs that may be useful for AD treatment are glucagon-like peptide-1 (GLP-1) agonists, involved in insulin release. Their ability to target insulin-resistance in AD while reducing neuroinflammation, oxidative stress, and ameliorating canonical AD proteinopathies makes them a prime therapeutic candidate for AD, reviewed previously.[16] They may also have benefits for sleep-disorders, specifically OSA, providing an additional therapeutic option for

concomitant dementia and OSA. Individuals with OSA have altered levels of serum GLP-1 compared to healthy controls, although the directionality is inconsistent.[17–19] Furthermore, acute sleep deprivation can alter postprandial circulating GLP-1 dynamics in men.[20,21] Benefits of these GLP-1 agonists has been shown in REM sleep-deprived rats, as treatment reduced SD-associated memory impairments and elevated Calcium/calmodulin-dependent protein kinase II, important for neuronal health.[22]

The relationship between sleep and AD extends into diagnostic biomarkers for AD as well. Accordingly, studies have shown an increased CSF tau/Aβ42 ratio with reduced NREM slow-wave sleep in AD patients.[23] In patients with OSA, a plethora of AD biomarkers may be altered including increased plasma Aβ42/Aβ40 ratio, total tau, and phospho-tau levels.[24,25] Other AD hallmarks including β-amyloid uptake in the cingulate gyrus and temporal cortex, reductions in overall gray matter volume in the brain, and memory impairments have all been linked to OSA.[26–28] It is important to note that not all biomarkers are homogenous across population demographics. For example, an interaction between OSA and the ApoE4 risk factor exists in older black/African American individuals, but not in white populations, and is associated with hippocampal volume.[29] This highlights the heterogeneity amongst ethnic groups and demonstrates the importance of inclusion of underrepresented populations. Finally, EEG is a useful digital biomarker to reliably monitor waves which correspond to AD development, including reduced alpha and beta waves with an associated uptick in theta and delta waves during wake.[30] Likewise, total tau levels negatively correlate with alpha power in males with MCI, but was not observed in females.[31] Another study found the slow-to-fast-activity ratio of entropy measured in REM sleep by EEG was able to differentiate AD dementia from MCI and controls.[32] Interestingly, at home sleep EEG may prove to be a useful digital biomarker for memory-related neural circuitry.[33]

Treatment	Main Pathway	Status	NCT Number	Phase	Results
Cannabidiol	Cannabinoid	Recruiting	NCT05822362	2	Not Available
Citicoline	Cholinergic	Active	NCT05200208	N/A	Not Available
		Active	NCT06029894	N/A	Not Available
Daridorexant	Orexinergic	Not Yet Recruiting	NCT07213349	2	Not Available
		Recruiting	NCT05924425	4	Not Available
Donepezil	Cholinergic	Completed	NCT00750529	1	Not Available
		Completed	NCT00480870	4	Cognitive improvement (ADAS-cog). Increased REM sleep percent (EEG).
Escitalopram	Serotonergic	Completed	NCT01841125	4	Not Available
Gabapentin Enacarbil	GABAergic	Recruiting	NCT03082755	4	Not Available
Galantamine	Cholinergic	Completed	NCT00750529	1	Not Available
		Completed	NCT00253227	3	Cognitive improvement (ADAS-cog). No impact on sleep quality (PSQI).
		Completed	NCT00035204	4	Not Available
GSK239512	Histaminergic	Completed	NCT01009255	2	Not Available
Lemborexant	Orexinergic	Recruiting	NCT06274528	2	Not Available
		Completed	NCT03001557	2	Inconsistent sleep improvement (actigraphy).
Melatonin (Circadin) with AChE inhibitor + Memantine	Melatonergic, Cholinergic, Glutamatergic	Completed	NCT00940589	2	Cognitive improvement (MMSE). Improved sleep efficiency (PSQI).
Melatonin	Melatonergic	Completed	NCT00000171	3	Not Available
		Completed	NCT03066518	4	No cognitive effects (MMSE). No impact on sleep quality (PSQI).
		Not Yet Recruiting	NCT06756828	N/A	Not Available
Mevidalen	Dopaminergic	Recruiting	NCT06538116	2	Not Available
Piromelatine	Melatonergic, Serotonergic	Completed	NCT02615002	2	No cognitive effects (cNTB, ADAS-cog). No impact on sleep quality (PSQI).
Ramelteon	Melatonergic	Completed	NCT00325728	2	No change in nighttime sleep (actigraphy).
Risperidone	Dopaminergic, Serotonergic	Completed	NCT00208819	4	Not Available

Suvorexant	Orexinergic	Recruiting	NCT04629547	2	Not Available
		Completed	NCT02750306	3	No cognitive effects (MMSE).<br>Increased total sleep time (PSG).
Trazadone	Serotonergic	Recruiting	NCT05282550	2	Not Available
		Completed	NCT01142258	3	No cognitive effects.<br>Improved nighttime sleep (actigraphy).
Zolpidem	GABAergic	Completed	NCT03075241	3	Worsened cognition (WAIS).<br>Improved nighttime sleep (actigraphy).
Zopiclone	GABAergic	Completed	NCT03075241	3	Worsened cognition (WAIS).<br>Improved nighttime sleep (actigraphy).

Supplementary Table 1: List of clinical trials targeting or measuring sleep in AD. ClinicalTrials.gov was searched using terms "Alzheimer" and "Sleep" to find studies where sleep quality or time is an outcome measure in AD or a sleep-promoting drug was used. Studies which had their status listed as "Unknown", "Terminated", or "Withdrawn" were not included. Abbreviations: ADAS-cog – Alzheimer's Disease Assessment Scale-Cognitive Subscale; cNTB – Computerized Neuropsychological Test Battery; EEG – Electroencephalogram; MMSE – Mini-Mental State Examination; PSQI – Pittsburgh Sleep Quality Index; WAIS – Wechsler Adult Intelligence Scale.

References

1. Lleó, A., Greenberg, S. M. & Growdon, J. H. Current pharmacotherapy for Alzheimer's disease. *Annu Rev Med* **57**, 513–533 (2006).
2. Howard, R. *et al.* Donepezil and memantine for moderate-to-severe Alzheimer's disease. *N Engl J Med* **366**, 893–903 (2012).
3. Grossberg, G. T. *et al.* The safety, tolerability, and efficacy of once-daily memantine (28 mg): a multinational, randomized, double-blind, placebo-controlled trial in patients with moderate-to-severe Alzheimer's disease taking cholinesterase inhibitors. *CNS Drugs* **27**, 469–478 (2013).
4. Song, M. S., Rauw, G., Baker, G. B. & Kar, S. Memantine protects rat cortical cultured neurons against beta-amyloid-induced toxicity by attenuating tau phosphorylation. *Eur J Neurosci* **28**, 1989–2002 (2008).
5. van Dyck, C. H. *et al.* Lecanemab in Early Alzheimer's Disease. *N Engl J Med* **388**, 9–21 (2023).
6. Lucey, B. P. & Musiek, E. S. Potential of dual orexin receptor antagonists to prevent Alzheimer's disease. *Alzheimers Dement* **20**, e090830 (2025).
7. Eisai Inc. *A Multicenter, Randomized, Double-Blind, Placebo-Controlled, Parallel-Group Study With Open-Label Extension Phase of the Efficacy and Safety of Lemborexant in Subjects With Irregular Sleep-Wake Rhythm Disorder and Mild to Moderate Alzheimer's Disease Dementia*. https://clinicaltrials.gov/study/NCT03001557 (2021).
8. Herring, W. J. *et al.* Polysomnographic assessment of suvorexant in patients with probable Alzheimer's disease dementia and insomnia: a randomized trial. *Alzheimers Dement* **16**, 541–551 (2020).

9. Doghramji, K. Melatonin and Its Receptors: A New Class of Sleep-Promoting Agents. *J Clin Sleep Med* **3**, S17–S23 (2007).

10. Sharma, R., Sahota, P. & Thakkar, M. M. Melatonin promotes sleep in mice by inhibiting orexin neurons in the perifornical lateral hypothalamus. *J Pineal Res* **65**, e12498 (2018).

11. Morales-Delgado, R. *et al.* A randomized placebo-controlled trial evaluating the effect of melatonin on sleep quality in patients with mild–moderate dementia. *Eur Geriatr Med* **9**, 449–454 (2018).

12. Takeda. *A Double-Blind, Randomized, Placebo-Controlled Study of the Efficacy, Safety and Tolerability of 8 Week Treatment of Rozerem 8 Mg (QHS) in Sleep Disturbed, Mild to Moderately Severe Alzheimer's Disease Subjects*. https://clinicaltrials.gov/study/NCT00325728 (2017).

13. Murillo-Rodríguez, E. The role of the CB1 receptor in the regulation of sleep. *Progress in Neuro-Psychopharmacology and Biological Psychiatry* **32**, 1420–1427 (2008).

14. Pees, A. *et al.* Sex- and age-specific sensitivities of the endocannabinoid system in Alzheimer's disease revealed by PET imaging with [^{18}F]FMPEP-d_2 and [^{18}F]MAGL-2102. *Theranostics* **15**, 3368–3385 (2025).

15. Louzada, L. L. *et al.* The efficacy and safety of zolpidem and zopiclone to treat insomnia in Alzheimer's disease: a randomized, triple-blind, placebo-controlled trial. *Neuropsychopharmacology* **47**, 570–579 (2022).

16. Du, H., Meng, X., Yao, Y. & Xu, J. The mechanism and efficacy of GLP-1 receptor agonists in the treatment of Alzheimer's disease. *Front Endocrinol (Lausanne)* **13**, 1033479 (2022).

17. Xu, S. *et al.* Efficacy of Glucagon-Like Peptide-1 Receptor Agonists in Obese or Diabetic Patients With Obstructive Sleep Apnea Syndrome: A Meta-Analysis. *Nutr Rev* nuaf115 (2025) doi:10.1093/nutrit/nuaf115.
18. Gonnissen, H. K. J., Hursel, R., Rutters, F., Martens, E. A. P. & Westerterp-Plantenga, M. S. Effects of sleep fragmentation on appetite and related hormone concentrations over 24 h in healthy men. *Br J Nutr* **109**, 748–756 (2013).
19. Matsumoto, T. *et al.* Plasma Incretin Levels and Dipeptidyl Peptidase-4 Activity in Patients with Obstructive Sleep Apnea. *Ann Am Thorac Soc* **13**, 1378–1387 (2016).
20. Benedict, C., Barclay, J. L., Ott, V., Oster, H. & Hallschmid, M. Acute sleep deprivation delays the glucagon-like peptide 1 peak response to breakfast in healthy men. *Nutr Diabetes* **3**, e78 (2013).
21. Gil-Lozano, M. *et al.* Short-term sleep deprivation with nocturnal light exposure alters time-dependent glucagon-like peptide-1 and insulin secretion in male volunteers. *Am J Physiol Endocrinol Metab* **310**, E41-50 (2016).
22. Turan, I., Sayan Ozacmak, H., Ozacmak, V. H., Ergenc, M. & Bayraktaroğlu, T. The effects of glucagon-like peptide 1 receptor agonist (exenatide) on memory impairment, and anxiety- and depression-like behavior induced by REM sleep deprivation. *Brain Res Bull* **174**, 194–202 (2021).
23. Lucey, B. P. *et al.* Reduced non-rapid eye movement sleep is associated with tau pathology in early Alzheimer's disease. *Sci Transl Med* **11**, eaau6550 (2019).
24. Liu, Y. *et al.* Sleep duration and efficiency are associated with plasma amyloid-β in non-demented older people. *Neurol Sci* **43**, 305–311 (2022).

25. Kong, W., Zheng, Y., Xu, W., Gu, H. & Wu, J. Biomarkers of Alzheimer’s disease in severe obstructive sleep apnea-hypopnea syndrome in the Chinese population. *Eur Arch Otorhinolaryngol* **278**, 865–872 (2021).
26. Ferini-Strambi, L. *et al.* Cognitive dysfunction in patients with obstructive sleep apnea (OSA): partial reversibility after continuous positive airway pressure (CPAP). *Brain Res Bull* **61**, 87–92 (2003).
27. Cross, N. *et al.* Is Obstructive Sleep Apnoea Related to Neuropsychological Function in Healthy Older Adults? A Systematic Review and Meta-Analysis. *Neuropsychol Rev* **27**, 389–402 (2017).
28. Liguori, C. *et al.* Sleep-disordered breathing and the risk of Alzheimer’s disease. *Sleep Med Rev* **55**, 101375 (2021).
29. Turner, A. D. *et al.* Exploring the combined effects of sleep apnea and APOE-e4 on biomarkers of Alzheimer’s disease. *Front Aging Neurosci* **14**, 1017521 (2023).
30. Malek, N., Baker, M. R., Mann, C. & Greene, J. Electroencephalographic markers in dementia. *Acta Neurol Scand* **135**, 388–393 (2017).
31. Chino-Vilca, B. *et al.* Sex specific EEG signatures associated with cerebrospinal fluid biomarkers in mild cognitive impairment. *Clin Neurophysiol* **142**, 190–198 (2022).
32. Azami, H. *et al.* EEG entropy in REM sleep as a physiologic biomarker in early clinical stages of Alzheimer’s disease. *J Alzheimers Dis* **91**, 1557–1572 (2023).
33. Pulver, R. L. *et al.* Mapping sleep’s oscillatory events as a biomarker of Alzheimer’s disease. *Alzheimers Dement* **20**, 301–315 (2023).
